# Determinants of intention to conceal tuberculosis status among family members: an analysis of seven Sub-Saharan African countries

**DOI:** 10.1186/s12879-024-09064-y

**Published:** 2024-02-08

**Authors:** William Dormechele, Emmanuel Osei Bonsu, Caleb Boadi, Mercy Oseiwah Adams, Benedictus Atsu Hlormenu, Stephen Kwakye Addo, Bright Boatey Bossman, Isaac Yeboah Addo

**Affiliations:** 1 Navrongo Health Research Centre, Upper East Region, Navrongo, Ghana; 2 Department of Epidemiology and Biostatistics, Kwame Nkrumah University of Science and Technology, Kumasi, Ghana; 3 Department of Operations and Management Information Systems, University of Ghana, Accra, Ghana; 4 Asesewa Government Hospital, Ghana Health Service, Asesewa, Ghana; 5 Ho, Ghana; 6 Accra, Ghana; 7 Information Technology Directorate, Aspire International School, Accra, Ghana; 8 Centre for Social Research in Health, University of New South Wales, Sydney, NSW Australia

**Keywords:** TB disclosure, Health secrecy, Stigma, Family health, Infectious disease, Africa

## Abstract

**Background:**

Tuberculosis (TB) remains a significant public health burden in Sub-Saharan Africa (SSA), accounting for about 25% of global TB cases. In several communities, TB diagnosis, treatment, and control have become a critical challenge, largely due to the intention to conceal TB status among family members. It is therefore crucial to understand the factors associated with the intentions to conceal TB status among family members in SSA.

**Methods:**

This quantitative study utilised data from the most recent Demographic and Health Surveys (DHS). The objective was to examine the factors associated with the intention to conceal the TB status of family members. The sample consisted of 58,849 individuals aged 10 years or older from seven SSA countries. Binary logistic regression was employed to assess the associations between TB status concealment and various socio-demographic and economic variables.

**Results:**

The overall prevalence of TB status concealment intentions for the seven countries was 28.0% (95% CI: 27.6–28.4). Malawi and Eswatini accounted for the highest (47.3%) and lowest (3.0%) prevalence of TB concealment intentions respectively. TB status concealment intentions decreased with increasing age (*p* < 0.001). Living in rural areas was associated with lower odds of intending to conceal the TB of family members compared to living in urban areas (aOR = 0.92; *p* = 0.008). Higher education levels were associated with lower odds of TB status concealment intentions (aOR = 0.50; *p* < 0.001) compared to lower education levels. As participants wealth index increased, the odds of TB status concealment intentions decreased (aOR = 0.83; *p* < 0.001). Country of residence also showed significant associations with individuals in Ghana (aOR = 4.51; *p* < 0.001), Lesotho (aOR = 2.08; *p* < 0.001), Malawi (aOR = 4.10; *p* < 0.001), Namibia (aOR = 4.40; *p* < 0.001), and Sao-Tome and Principe (aOR = 5.56; *p* < 0.001) showing higher odds of TB status concealment intentions compared to Eswatini.

**Conclusions:**

The findings conclude that several social determinants of health, including age, urbanicity, education, and wealth contribute to TB status concealment intentions for family members. Considering these factors is important for designing targeted interventions to improve TB control in the sample. In light of the unavailability of cultural variables in the dataset, future research can leverage qualitative approaches to conduct a more comprehensive exploration of the cultural factors linked to TB status concealment intentions in the population.

## Introduction

Tuberculosis (TB) continues to be a significant global health burden, particularly in Sub-Saharan Africa (SSA), where it remains a major public health challenge [1–3]. The World Health Organization (WHO) estimates that SSA alone accounts for a disproportionate 25% of the world’s TB cases [4]. TB transmission within and between households is a critical concern in the region, as individuals in close contact with TB patients are at a heightened risk of acquiring the infection and subsequently spreading it to others in the community [5]. This underscores the importance of understanding the involvement of family members in managing TB for affected individuals. Thus, family decisions and support have substantial ramifications for disease transmission and control, as well as the overall well-being and treatment results of TB patients [6].

Evidence shows that TB-related stigma is pervasive in several SSA communities, driving individuals to hide their TB status to avoid discrimination and isolation [7]. The fear of social “ostracization” can heavily influence a family or individual’s choice to conceal their TB status or the status of their family members, thereby impeding contact tracing efforts and TB case identification within and between households [8]. This can lead to delayed diagnosis, delayed treatment initiation, and further transmission of the disease, as a result, exacerbating the burden of the disease [9]. It is therefore essential to develop comprehensive strategies that focus on destigmatising TB and promoting open communication about the disease. By creating supportive environments, individuals may feel more comfortable disclosing their TB status, seeking timely diagnosis and treatment, and ultimately contributing to better overall TB control in the region.

Intention to conceal the TB status of family members is crucial to the disease’s spread and control and may involve individuals intentionally hiding their TB symptoms or diagnosis from their relatives and the community, or family members deciding to keep the TB status of a family member a secret from the community [10, 11]. While evidence shows that these behaviours might stem from the fear of stigmatisation and discrimination [10], it is crucial to investigate the extent to which various social determinants are linked to these decisions. Investigating the social determinants associated with family decisions to conceal TB status holds significant importance for several reasons. Firstly, comprehending the underlying socio-economic factors driving TB concealment can enable the design of targeted interventions to address these issues effectively. By addressing the root causes, public health initiatives can enhance TB transmission control and improve treatment outcomes. Secondly, socio-economic disparities can create vulnerable populations more inclined to conceal TB status. Identifying these at-risk groups can assist healthcare providers and policymakers in allocating resources and customising interventions to cater to those most in need. Lastly, understanding the socio-economic context can empower healthcare providers to deliver patient-centred care, acknowledging the distinct challenges and requirements of individuals and families impacted by TB.

Despite the significance of understanding the social determinants associated with intentions to conceal TB status in SSA, limited research has been conducted on this topic. The closest study to date was carried out in Ghana approximately seven years ago [10]. The scarcity of previous research on this topic, the considerable time gap since the nearest study, and the absence of studies encompassing variables across multiple countries emphasise the urgent need for an extensive investigation into the social determinants that drive individuals’ choices to conceal their TB status in SSA. Such research will be critical for unveiling nuanced insights into the multifaceted socio-cultural, economic, and health-related factors that underpin TB status concealment in the region. This study, therefore, aimed to examine the socio-demographic and economic factors associated with the intention to keep TB status concealed among family members in seven SSA countries using two response categories- “Yes” or “No”. Based on the evidence in several studies [11–14], we hypothesised that the intention to conceal TB status among family members would demonstrate significant associations with variables, such as age, sex, marital status, place of residence, education, religion, exposure to media, and wealth. Our approach offers two significant contributions to the existing literature. Firstly, it enhances the methodological approach utilised in the previous research [10]. Secondly, it expands the research scope from a single country to encompass seven countries with relevant data. Unravelling these complexities holds the key to targeted interventions, support systems, and stigma reduction strategies that can transform TB management and care in the included countries.

## Methods

### Study design and data source

This quantitative study employed nationally representative data from seven SSA countries collected through the Demographic and Health Surveys (DHS). Written approval was obtained from the DHS program to use their dataset for the study (Appendix 1: approval letter). The research aimed to gain insights into TB concealment among family members.

### Overview of the DHS and data collection

The Demographic and Health Survey (DHS) is a comprehensive and nationally representative data collection program that aims to provide vital health and demographic information. The DHS data collection for TB is an integral part of this program and plays a crucial role in understanding the prevalence and factors associated with TB in various countries, including those within SSA [15, 16].

The DHS data collection for TB focuses on capturing information related to the occurrence, management, and impact of TB in specific SSA countries. The program is designed to cover a diverse range of households and communities to ensure a representative sample of the population [16].

The DHS administers standardised questionnaires that are adapted to capture detailed information about TB-related indicators. These questionnaires are designed to gather data from household members, including demographic characteristics, health status, and TB-related knowledge, attitudes, and practices [16].

As part of the data collection, DHS include TB testing and diagnosis in selected households. This may involve collecting sputum samples from individuals with symptoms suggestive of TB and testing for the presence of *Mycobacterium tuberculosis* using appropriate diagnostic methods, such as microscopy or GeneXpert [16]. The DHS also assesses the knowledge and awareness of TB among household members. This includes the understanding of TB symptoms, modes of transmission, and available treatment options [16].

The data collection includes the gathering of socio-economic information, such as education, occupation, and household wealth index. These factors can influence TB risk and impact access to healthcare services [16].

### Process for accessing DHS program data

Accessing datasets within the DHS Program involves navigating to the program’s website and selecting the desired survey or dataset. To initiate access, individuals must register for an account and complete a data access request form as per the provided instructions. Following approval, datasets can be downloaded for utilisation exclusively within the scope of the intended research or study. Should there be a need for additional or alternative data for distinct research purposes, a separate research project request must be submitted anew.

### Inclusion and exclusion criteria

The study comprised individuals aged 10 years and older. Specifically, countries with the outcome variable of interest (keeping secret when a family member gets TB) were included, while those without the outcome variable were excluded. From the DHS data obtained from thirty-nine SSA countries on 4th May 2023, seven countries (Eswatini 2006/07, Ghana 2014, Lesotho 2014, Liberia 2019/20, Malawi 2015/16, Namibia 2013, and Sao-Tome and Principe 2008/09) met the inclusion criteria and were considered for analysis [17].

### Sampling and sample size

The DHS sampling design utilised a stratified two-stage cluster approach. In the first stage, Enumeration Areas (EA) were drawn from Census files. In the second stage, households were selected from an updated list within each chosen EA. As census data from the DHS database were used, the sample size was not calculated. The study retained a total of 58,849 cases from seven SSA countries for analysis.

### Dependent variable

The dependent variable for this study was derived from the question which asked survey participants whether they would “keep secret when a family member gets TB”, with three response options - “no = 0”, “yes = 1” and “don’t know/not sure/depends = 8”. In this study, however, we maintained “[No = 0]” and “[Yes = 1] to generate a binary variable and dropped the 2.68% of “don’t know/not sure/depends” responses [10, 18–20]. We contend that “No” is a clear denial or negation, whereas “don’t know/not sure/depends” are an admission of not having the necessary information or knowledge on the subject [19, 20]. “No” implies a clear understanding or awareness of the question or statement being asked, and the respondent knows that the answer is negative. On the other hand, “Don’t Know” indicates a lack of knowledge or uncertainty about the question and the respondent is unable to provide a definitive response. Therefore, treating “Don’t Know” as “No” would lead to incorrect data interpretation and may introduce bias into the analysis. To ensure data accuracy, it is crucial to treat “Don’t Know” as a distinct response category. By doing so, researchers can avoid misinterpreting the data and obtain more reliable and meaningful results. Therefore, unlike a previous study − [10] “Don’t Know” was not considered synonymous with “No” in this study.

### Independent variables

Based on a review of the existing literature [11–14], various independent variables were used for analysis. Age groups were categorised into five groups: 10–19 years = 1, 20–29 years = 2, 30–39 years = 3, 40–49 years = 4, and ≥ 50 years = 5. Sex was categorised as male = 1 and female = 2. Marital status was divided into never married = 0, married = 1, living together = 2, widowed = 3, and divorced = 4. Education level was classified as no education = 0, primary = 1, secondary = 2, and higher = 3. The household wealth index was categorised into five groups: poorest = 1, poorer = 2, middle = 3, richer = 4, and richest = 5. The place of residence was divided into urban = 1 and rural = 2. Religion was categorised as Christian = 1, Islam = 2, and others = 3. Countries were categorised based on specific names: Eswatini = 1, Ghana = 2, Lesotho = 3, Liberia = 4, Malawi = 5, Namibia = 6, and Sao-Tome and Principe = 7. Exposure to media sources was classified based on frequency: not at all = 0, less than once a week = 1, at least once a week = 2, and nearly every day = 3, including exposure to media such as radio, newspapers/magazines, and television. These independent variables were utilised in logistic regression analysis to explore their associations with the outcome variable of interest - keeping secrets when a family member gets TB.

### Statistical analysis

The data was prepared for survey analysis using svyset to account for the complex sampling nature. Descriptive analysis and binary logistic regression were then performed to analyse the data. The initial step involved conducting descriptive bivariate analysis to examine the relationships between the independent variables listed earlier. Frequencies, percentages, means, maximums, and minimums were computed to provide summary measures and descriptions of the fundamental characteristics of the data. Additionally, the corresponding Chi-square test results were presented. As the outcome variables were dichotomous (represented by ‘1’ and ‘0’ for yes/no responses regarding keeping a secret when a family member gets TB), binary logistic regression models were used. We applied weighting as “sample weight” variable divided by 1,000,000. Cluster design was applied using the command: svyset [pweight = wgt], strata(sample_stratum_num) vce(linearised) singleunit(centered). A p-value of less than 0.05 with a 95% confidence level was considered statistically significant, and adjusted odds ratios were used to assess the strength of associations. All estimations were conducted using the svy command in STATA version 18.

### Ethical consideration

The study received written approval from the DHS Program to utilise their datasets. The procedures for using DHS public-use datasets, approved by the Institutional Review Board (IRB), ensure that the identity of respondents, households, and sample communities remains protected and cannot be identified. The data files did not contain any names of individuals or household addresses. Permission was obtained from http://www.dhsprogram.com to download and use the data specifically for this study.

## Results

### Participant characteristics

The results regarding respondents’ characteristics are presented in Table 1. The weighted mean age of the study participants was 28.9 years. The age group with the highest representation among the participants was 20–29 years, accounting for 36.3% (95% CI: 35.9–36.7%). Approximately 60.8% (95% CI: 60.4–61.2%) of household heads were male. Most participants were married, comprising 43.6% (95% CI: 43.2–44.0%) of the sample. The weighted data indicates that 61.4% (95% CI: 61.0–61.8%) of the participants resided in rural areas. Furthermore, the most common educational level was secondary education, representing 42.7% (95% CI: 42.3–43.1%) of participants. Similarly, most participants followed the Christian religion, comprising 93.9% (95% CI: 93.7–94.1%) of the sample. Regarding media exposure, majority of the participants (66.3%, 95% CI: 65.9–66.6%) did not read newspapers at all but listened to the radio at least once a week (39.2%, 95% CI: 38.8–39.5%). About 25.1% of the participants (95% CI: 24.8–25.5%) were in the “richest” wealth category (Table 1).

**Table 1:** Background characteristics of study participants

Characteristics	Unweighted, (*N* = 58,682)		Weighted, (*N* = 58,849)	
	n (%)	Logit [95 CI]	%	Logit [95 CI]
**Age in years [mean(sd)]**	28.9 (9.5)	28.8–29.0	28.8 (9.5)	28.7–28.9
**Age in years**				
10–19	11,821 (20.1)	19.8–20.5	19.9	19.6–20.2
20–29	20,886 (35.6)	35.2–36	36.3	35.9–36.7
30–39	15,757 (26.9)	26.5–27.2	26.8	26.5–27.2
40–49	10,218 (17.4)	17.1–17.7	17.0	16.7–17.3
**Sex of participants**				
Male	36,238 (61.8)	61.4–62.1	60.8	60.4–61.2
Female	22,444 (38.2)	37.9–38.6	39.2	38.8–39.6
**Current marital status**				
Never married	18,922 (32.2)	31.9–32.6	33.5	33.1–33.9
Married	25,938 (44.2)	43.8–44.6	43.6	43.2–44.0
Living together	7,232 (12.3)	12.1–12.6	11.8	11.6–12.1
Widowed	1,950 (3.3)	3.2–3.5	3.2	3.1–3.4
Divorced	4,640 (7.9)	7.7–8.1	7.9	7.7–8.1
**Type of place of residence**				
Urban	20,965 (35.7)	35.3–36.1	38.6	38.2–39
Rural	37,717 (64.3)	63.9–64.7	61.4	61.0–61.8
**Educational level**				
No formal education	7,428 (12.7)	12.4–12.9	11.3	11–11.5
Primary	24,108 (41.1)	40.7–41.5	40.0	39.6–40.4
Secondary	24,229 (41.3)	40.9–41.7	42.7	42.3–43.1
Higher	2,917 (5.0)	4.8–5.1	6.0	5.8–6.2
**Religion**				
Christian	54,936 (93.6)	93.4–93.8	93.9	93.7–94.1
Islam	2,164 (3.7)	3.5–3.8	3.3	3.2–3.5
Other	1,582 (2.7)	2.6–2.8	2.8	2.7–2.9
**Frequency of reading newspaper**				
Not at all	39,471 (67.3)	66.9–67.6	66.3	65.9–66.6
Less than once a week	9,403 (16.0)	15.7–16.3	16.4	16.1–16.7
At least once a week	8,246 (14.1)	13.8–14.3	14.8	14.5–15.1
Almost every day	1,562 (2.7)	2.5–2.8	2.5	2.4–2.7
**Frequency of listening to a radio**				
Not at all	19,444 (33.1)	32.8–33.5	32.6	32.2–32.9
Less than once a week	12,156 (20.7)	20.4–21	21.4	21–21.7
At least once a week	22,958 (39.1)	38.7–39.5	39.2	38.8–39.5
Almost every day	4,124 (7.0)	6.8–7.2	6.9	6.7–7.1
**Frequency of watching television**				
Not at all	34,698 (59.1)	58.7–59.5	57.9	57.5–58.2
Less than once a week	7,639 (13.0)	12.7–13.3	13.5	13.2–13.8
At least once a week	13,699 (23.3)	23–23.7	24.2	23.9–24.6
Almost every day	2,646 (4.5)	4.3–4.7	4.4	4.2–4.6
**Wealth index**				
Poorest	10,517 (17.9)	17.6–18.2	16.1	15.8–16.4
Poorer	10,912 (18.6)	18.3–18.9	17.6	17.3–17.9
Middle	11,510 (19.6)	19.3–19.9	19.1	18.8–19.5
Richer	12,199 (20.8)	20.5–21.1	22.1	21.7–22.4
Richest	13,544 (23.1)	22.7–23.4	25.1	24.8–25.5
**Country**				
Eswatini	4,692 (8.0)	7.8–8.2	8.0	7.8–8.2
Ghana	7,402 (12.6)	12.3–12.9	12.9	12.6–13.2
Lesotho	6,235 (10.6)	10.4–10.9	10.6	10.4–10.9
Liberia	7,247 (12.3)	12.1–12.6	12.4	12.1–12.6
Malawi	22,783 (38.8)	38.4–39.2	38.6	38.2–39
Namibia	8,406 (14.3)	14.0–14.6	14.4	14.1–14.6
Sao-Tome and Principe	1,917 (3.3)	3.1–3.4	3.1	3.0–3.3

### Bivariate analysis of determinants of the intention to conceal TB status of family members

The bivariate analysis examined factors influencing the intention to conceal the TB status of family members. It revealed several statistically significant associations (Table 2). The age distribution of participants with the intentions to conceal the TB status of family members was significantly different from those without the intention to conceal (p-value < 0.001), suggesting evidence against the null hypothesis. Participants’ highest attained educational level showed a significant difference for those with the intention to conceal the TB status of a family member and those without the intention to conceal (p-value < 0.001), supporting evidence against the null hypothesis. The frequency of watching television showed significant associations with the intention to conceal the TB status of family members (p-value < 0.001), providing evidence against the null hypothesis. (Table 2).

**Table 2:** Bivariate analysis of factors associated with the Intention to conceal TB Status in Family Members in SSA

Characteristics	Intention to conceal TB Status		P-value
	No, n (%)	Yes, n (%)	
	(*N* = 42,251)	(*N* = 16,431)	
Current age (Continuous)	29.41 (9.55)	27.56 (9.38)	< 0.001
**Current age (Categorical)**			
10–19	7,753 (18.3)	4,068 (24.8)	< 0.001
20–29	14,857 (35.2)	6,029 (36.7)	
30–39	11,758 (27.8)	3,999 (24.3)	
40–49	7,883 (18.7)	2,335 (14.2)	
**Sex of participants**			
Male	25,911 (61.3)	10,327 (62.9)	< 0.001
Female	16,340 (38.7)	6,104 (37.1)	
**Current marital status**			
Never married	13,370 (31.6)	5,552 (33.8)	< 0.001
Married	18,619 (44.1)	7,319 (44.5)	
Living together	5,438 (12.9)	1,794 (10.9)	
Widowed	1,489 (3.5)	461 (2.8)	
Divorced	3,335 (7.9)	1,305 (7.9)	
**Type of place of residence**			
Urban	15,538 (36.8)	5,427 (33.0)	< 0.001
Rural	26,713 (63.2)	11,004 (67.0)	
**Educational level**			
No formal education	5,447 (12.9)	1,981 (12.1)	< 0.001
Primary	16,462 (39.0)	7,646 (46.5)	
Secondary	17,915 (42.4)	6,314 (38.4)	
Higher	2,427 (5.7)	490 (3.0)	
**Religion**			
Christian	39,624 (93.8)	15,312 (93.2)	< 0.001
Islam	1,443 (3.4)	721 (4.4)	
Other	1,184 (2.8)	398 (2.4)	
**Frequency of reading newspaper**			
Not at all	27,936 (66.1)	11,535 (70.2)	< 0.001
Less than once a week	6,726 (15.9)	2,677 (16.3)	
At least once a week	6,263 (14.8)	1,983 (12.1)	
Almost every day	1,326 (3.1)	236 (1.4)	
**Frequency of listening to a radio**			
Not at all	13,707 (32.4)	5,737 (34.9)	< 0.001
Less than once a week	8,754 (20.7)	3,402 (20.7)	
At least once a week	16,347 (38.7)	6,611 (40.2)	
Almost every day	3,443 (8.1)	681 (4.1)	
**Frequency of watching television**			
Not at all	24,722 (58.5)	9,976 (60.7)	< 0.001
Less than once a week	5,530 (13.1)	2,109 (12.8)	
At least once a week	9,860 (23.3)	3,839 (23.4)	
Almost every day	2,139 (5.1)	507 (3.1)	
**Wealth index**			
Poorest	7,165 (17.0)	3,352 (20.4)	< 0.001
Poorer	7,802 (18.5)	3,110 (18.9)	
Middle	8,232 (19.5)	3,278 (20.0)	
Richer	8,881 (21.0)	3,318 (20.2)	
Richest	10,171 (24.1)	3,373 (20.5)	
**Country**			
Eswatini	4,205 (10.0)	487 (3.0)	< 0.001
Ghana	4,689 (11.1)	2,713 (16.5)	
Lesotho	4,929 (11.7)	1,306 (7.9)	
Liberia	6,370 (15.1)	877 (5.3)	
Malawi	15,014 (35.5)	7,769 (47.3)	
Namibia	5,762 (13.6)	2,644 (16.1)	
Sao-Tome and Principe	1,282 (3.0)	635 (3.9)	

*Statistically significant at *p* < 0.05; n represents total frequency

### Multivariate logistic regression results of factors associated with the intention to conceal TB status of family members

The results from the multivariate logistic regression analysis investigating the intention to conceal TB status among family members in SSA revealed several statistically significant associations. Firstly, increasing age was associated with decreased odds of intentions to conceal the TB status of family members (*p* < 0.001). Specifically, the odds were lower for the 20–29, 30–39, and 40–49 age groups compared to the 10–19 age group. Secondly, individuals living in rural areas had lower odds of intending to conceal the TB status of family members compared to those residing in urban areas (*p* = 0.008). Thirdly, higher education levels were linked to lower odds of intending to conceal the TB status of family members compared to individuals with no education (*p* < 0.001). Regarding religion, individuals following the Islamic faith had higher odds of intending to conceal the TB status of family members than Christians (*p* = 0.002), while individuals of other religions had lower odds of concealment intentions (*p* = 0.003).

Interestingly, individuals who read newspapers almost every day had higher odds of intending to conceal the TB status of family members compared to those who did not read at all (*p* = 0.133). Additionally, as the wealth index increased, the odds of intending to conceal the TB status of family members decreased (*p* < 0.001). Moreover, the country of residence exhibited significant associations with TB status concealment intentions. For instance, individuals in Ghana, Lesotho, Malawi, Namibia, and Sao-Tome and Principe had higher odds of intending to conceal the TB status of family members compared to Eswatini (all p-values < 0.001) (Table 3).

These findings highlight the various factors influencing the intention to conceal the TB status of family members in SSA, offering valuable insights for public health interventions and policy development in the region.

**Table 3:** Logistic regression of factors associated with the intention to conceal TB Status in family members in SSA

Factors	Univariate model			Multivariate model		
	OR	95% CI	p-value	aOR	95% CI	p-value
**Current age (Categorical)**						
10–19	1.00			1.00		
20–29	0.76	0.71–0.80	< 0.001	0.73	0.68–0.79	< 0.001
30–39	0.63	0.59–0.67	< 0.001	0.56	0.52–0.61	< 0.001
40–49	0.55	0.51–0.59	< 0.001	0.47	0.43–0.52	< 0.001
**Sex of participants**						
Male	1.00			1.00		
Female	0.94	0.89–0.98	0.005	1.03	0.97–1.08	0.363
**Current marital status**						
Never married	1.00			1.00		
Married	0.97	0.92–1.02	0.233	1.03	0.95–1.11	0.473
Living together	0.86	0.80–0.93	< 0.001	1.00	0.91–1.09	0.933
Widowed	0.76	0.66–0.87	< 0.001	1.04	0.89–1.21	0.601
Divorced	0.99	0.91–1.08	0.855	1.00	0.90–1.11	0.973
**Type of place of residence**						
Urban	1.00			1.00		
Rural	1.23	1.18–1.29	< 0.001	0.92	0.86–0.98	0.008
**Highest educational level**						
No education	1.00			1.00		
Primary	1.26	1.17–1.35	< 0.001	0.90	0.83–0.98	0.015
Secondary	0.91	0.84–0.98	0.009	0.70	0.64–0.77	< 0.001
Higher	0.54	0.47–0.62	< 0.001	0.50	0.42–0.58	< 0.001
**Religion**						
Christian	1.00			1.00		
Islam	1.13	1.01–1.27	0.034	1.22	1.07–1.38	0.002
Other	0.87	0.76–1.00	0.047	0.80	0.69–0.92	0.003
**Frequency of reading newspaper**						
Not at all	1.00			1.00		
Less than once a week	0.97	0.91–1.03	0.369	1.03	0.95–1.10	0.493
At least once a week	0.76	0.71–0.82	< 0.001	0.86	0.79–0.93	< 0.001
Almost every day	0.44	0.38–0.52	< 0.001	1.16	0.95–1.42	0.139
**Frequency of listening to a radio**						
Not at all	1.00			1.00		
Less than once a week	0.93	0.87–0.99	0.017	0.99	0.92–1.06	0.715
At least once a week	0.89	0.85–0.94	< 0.001	1.00	0.94–1.06	0.929
Almost every day	0.50	0.45–0.56	< 0.001	0.93	0.78–1.11	0.399
**Frequency of watching television**						
Not at all	1.00			1.00		
Less than once a week	0.91	0.85–0.98	0.009	1.00	0.92–1.08	0.999
At least once a week	0.88	0.83–0.93	< 0.001	0.95	0.88–1.02	0.171
Almost every day	0.66	0.59–0.75	< 0.001	0.87	0.72–1.06	0.163
**Wealth index**						
Poorest	1.00			1.00		
Poorer	0.85	0.79–0.91	< 0.001	0.86	0.80–0.92	< 0.001
Middle	0.84	0.78–0.90	< 0.001	0.89	0.83–0.96	0.004
Richer	0.73	0.68–0.78	< 0.001	0.83	0.77–0.90	< 0.001
Richest	0.68	0.63–0.73	< 0.001	0.84	0.76–0.93	< 0.001
**Country**						
Eswatini	1.00			1.00		
Ghana	4.25	3.79–4.77	< 0.001	4.50	3.82–5.29	< 0.001
Lesotho	2.10	1.86–2.37	< 0.001	2.07	1.76–2.44	< 0.001
Liberia	1.23	1.07–1.42	0.003	1.12	0.93–1.34	0.224
Malawi	4.56	4.11–5.06	< 0.001	4.08	3.50–4.76	< 0.001
Namibia	3.95	3.53–4.42	< 0.001	4.40	3.76–5.14	< 0.001
Sao-Tome and Principe	5.00	4.29–5.82	< 0.001	5.50	4.57–6.62	< 0.001

**OR**: Odds ratio; **aOR**: Adjusted Odds ratio; **CI**: Confidence interval

*Statistically significant at *p* < 0.05

## Discussion

The study aimed to examine the factors associated with the intention to conceal the TB status of family members. We found that more than a quarter of the sample (28%) admitted to intending to conceal the TB status of their family members. This elevated prevalence of TB status concealment intentions for family members highlights the necessity for targeted public health policies and interventions. Policymakers can prioritise enhancing community awareness and knowledge regarding TB, emphasising early diagnosis, treatment adherence, and transparent communication about TB status. Addressing the stigmatisation linked to TB is essential to foster an environment where individuals and families feel comfortable disclosing their TB status without fear of discrimination. By addressing these aspects, public health policies can play a pivotal role in mitigating TB status concealment intentions and improving TB control efforts.

Given the considerable prevalence of TB status concealment intentions for family members in this sample, it becomes imperative to explore the underlying factors driving such behaviours. Consistent with existing research that underscores the influence of socio-demographic factors on TB status concealment within households [10, 21–23], our findings reveal variations in TB status concealment intentions for family members based on participant’s age, education levels, residence, wealth status, and exposure to mass media. These associations shed light on the determinants of TB status concealment and emphasise the need for further investigation to inform targeted interventions and public health strategies.

More specifically, the findings show that increasing age is associated with a decreased likelihood of intentions to conceal the TB status of family members, which is consistent with a study conducted by Amo-Adjei [10] in Ghana, where older age groups were less likely to conceal their TB status. This pattern may be attributed to greater health awareness and responsibility among older individuals, leading to a higher likelihood of open communication regarding TB status [24]. As individuals age, they tend to accumulate more health-related experiences and knowledge [25], leading to heightened awareness of the importance of timely TB diagnosis and treatment. Consequently, older individuals may be more inclined to openly disclose the TB status of their family members to seek appropriate medical care. Moreover, their familiarity with TB, either through personal experiences or knowledge of affected family or friends, may reduce fear or stigma associated with the disease, making them more willing to share the TB status of their family members openly. Furthermore, older individuals often hold significant roles within their families, and concealing the TB status of their family members could adversely impact the health of their household [26]. Recognising the potential risks of TB transmission to loved ones, older individuals may prioritise open communication about the TB status of family members to safeguard their entire family’s well-being. Additionally, with age, individuals may develop a greater acceptance of health conditions and become more open about their health status [27], extending this acceptance to disclose the TB status of family members and contributing to the decreased likelihood of concealment. Lastly, older individuals may display reduced concern about social stigma related to TB compared to younger age groups due to having a more stable social network and established relationships [28], which mitigates fears of discrimination or social isolation if the TB status of other family members becomes known.

Education is a crucial factor in promoting health literacy and increasing awareness of the importance of early TB diagnosis and treatment [29]. The present study shows that individuals with higher education levels are more likely to understand the potential consequences of TB concealment and, therefore, may be more inclined to disclose the TB status of their family members. Similar to our findings, a study by Agho et al. in Nigeria [30] and Amo-Adjei in Ghana [10] also reported that higher educational attainment was associated with a reduced likelihood of concealing TB status. Higher education levels are often associated with improved health literacy [31], providing individuals with a deeper understanding of health conditions, such as TB and the importance of early detection and treatment. This heightened awareness may lead educated individuals to openly communicate about their TB status with their family members. Moreover, individuals with higher education levels typically have better access to information [32], healthcare resources, and medical services. This advantage may stem from formal health education or better access to reliable health information sources [32], influencing their inclination to disclose the TB status of family members. Additionally, higher education equips individuals with better communication skills [33], enabling them to express themselves effectively and discuss sensitive health issues like TB confidently with their family members. Furthermore, higher education can foster a broader perspective and reduce stigma associated with TB. Educated individuals often demonstrate open-mindedness and understanding of health conditions [34], making them more willing to share the TB status of their family members without fear of discrimination or social isolation. Lastly, higher education empowers individuals to take charge of their family’s health and advocate for their well-being [34]. Educated individuals may feel more empowered to openly discuss the TB status of their family members and proactively support them in seeking appropriate medical care.

Furthermore, the results demonstrate a notable association between living in rural areas and a decreased probability of TB status concealment intentions compared to urban areas. This finding of decreased probability of TB status concealment among people living in rural areas compared to urban areas can be influenced by multiple factors. First, rural communities tend to have closer-knit social networks and a stronger sense of community [35], fostering an environment of trust and support among neighbours and community members. This heightened sense of solidarity may encourage individuals to be more willing to openly disclose the TB status of family members. Moreover, in rural areas, the smaller population and closer social ties may limit opportunities for anonymity. As a consequence, concealing the TB status of family members becomes more challenging, as health-related information can spread quickly within the community, making it difficult to maintain secrecy. Furthermore, limited access to healthcare facilities and resources in rural areas may lead individuals to rely more heavily on their community for support and assistance [36]. As a result, there may be a greater emphasis on open communication about health issues, including TB, within rural settings. In contrast, urban settings can sometimes be more impersonal and anonymous [36], which might foster an environment where individuals feel more inclined to conceal their health conditions, including TB, to avoid potential stigmatisation. Additionally, differences in health literacy between rural and urban populations could also play a role [37]. In rural areas, individuals may have a better understanding of TB and its consequences, leading to greater awareness and openness about the condition [37]. Nevertheless, these findings contradict the outcomes of both Nyangoma et al.’s study [22] in Uganda and Nagarajan et al.’s study [8] in Chennai, where they reported a higher likelihood of TB status concealment among rural residents in comparison to those living in urban areas. This discrepancy could be attributed to variations in cultural norms or differences in the statistical power between the two study settings.

The study also revealed significant associations between religious affiliations and TB status concealment intentions, which is consistent with findings from Ngamvithayapong-Yanai et al. [38] in Thailand. Their research observed that specific religious beliefs, such as prayer and spirituality, and trust in God’s or Allah’s will, influenced health-seeking behaviours related to TB, potentially impacting the likelihood of concealing the TB status of family members. Health authorities and policymakers can devise awareness campaigns and educational initiatives targeted at specific religious beliefs linked to TB. Such efforts would aim to dispel misconceptions and foster open communication about TB within religious communities, thereby encouraging individuals to disclose their TB status more openly. Culturally sensitive healthcare delivery is essential, necessitating healthcare providers’ awareness of the cultural and religious context when discussing TB with patients and families. By emphasising the significance of early diagnosis and treatment while respecting religious beliefs, a supportive environment for TB disclosure and management can be cultivated. Engaging religious leaders in TB awareness and prevention endeavours can prove pivotal in reaching community members. Given their influential position within congregations, religious leaders’ support can promote health-seeking behaviours related to TB and combat the associated stigma. Additionally, the study highlights the importance of further research to investigate how religious beliefs influence TB-related health-seeking behaviours. Comparative studies across diverse regions and cultural contexts will deepen our understanding of the impact of religious affiliations on TB status concealment. This knowledge will contribute to more effective TB control strategies and culturally appropriate healthcare interventions.

Regarding wealth index, our findings align with previous research conducted in Malawi [21], Ethiopia [39], and Ghana [10] which reported that higher wealth index status was associated with a reduced TB status concealment. Individuals with greater financial resources may have better access to healthcare services, leading to early disclosing, and timely initiation of TB treatment. Individuals with higher financial resources are more likely to have improved access to healthcare facilities and services, enabling them to promptly seek medical attention for TB symptoms, leading to earlier diagnosis and treatment. This timely access to healthcare may reduce the need or motivation to conceal the TB status of family members. Moreover, higher wealth status is often associated with better health awareness and education. Those with greater financial resources tend to have a better understanding of the significance of early detection and treatment for TB, resulting in an increased willingness to openly discuss their TB status with family members. Furthermore, individuals with higher financial resources may have greater exposure to diverse social environments and interactions, potentially leading to reduced stigma associated with TB. The presence of a more inclusive and open-minded social circle may encourage individuals to disclose the TB status of family members without fear of discrimination. In addition, higher wealth status facilitates access to supportive networks and resources, including family, friends, or organisations that promote health-seeking behaviours and discourage concealing TB status. Moreover, greater financial resources empower individuals to make informed decisions about their health. This empowerment may lead to a stronger sense of control over their health outcomes and a greater willingness to openly communicate about the TB status of family members. Additionally, higher wealth status is often linked to better living conditions, including access to clean environments and adequate healthcare facilities. These improved living conditions can contribute to overall better health, reducing the need for individuals to hide the TB status of their family members.

Our findings support prior research [40] that found a link between mass media exposure and TB status concealment. The connection between mass media exposure and TB status concealment intentions can be attributed to the pervasive influence of media platforms in shaping societal perceptions, norms, and behaviours [41, 42]. Mass media, through its extensive reach and diverse communication channels, often disseminates information, attitudes, and beliefs about health-related matters, including infectious diseases like tuberculosis [43]. Exposure to media messages, whether through television, radio, social media, or other mediums, can significantly impact individuals’ understanding and attitudes toward TB [43]. Misconceptions, stigma, and fear associated with TB may be perpetuated or reinforced by media portrayals, potentially leading individuals to feel compelled to hide the TB status of family members due to concerns about discrimination, social isolation, or other negative consequences [44]. Furthermore, media campaigns or coverage that lacks accurate information or promotes stigmatising narratives about TB might contribute to fostering an environment where individuals feel inclined to conceal the TB status of their family members as a coping mechanism, influenced by the societal perceptions perpetuated through media exposure [40, 45]. People’s understanding and behaviours surrounding TB are influenced by media exposure, perhaps lowering stigma and boosting disclosure within families [40, 45]. Campaigns in the media aim to de-stigmatise TB, promote early diagnosis and treatment adherence, and underline the importance of reporting TB status for optimal disease management [44]. To this end, our study showed a link between mass media exposure and TB status concealment intentions, emphasising the significance of this component. However, further study is needed to evaluate how the media influences TB disclosure behaviours.

In terms of country-specific variations, our study found significant disparities in TB status concealment across different countries in SSA. Individuals in Ghana, Lesotho, Malawi, Namibia, and Sao-Tome and Principe had higher odds of intentions to conceal the TB status of family members compared to Eswatini. These findings suggest that contextual factors and country-specific healthcare policies may influence TB disclosure practices within households. This result corroborates with past research on cultural epidemiology [46], which found that long-standing community beliefs about a given disease are the source of regional variability in community perceptions of that disease.

Overall, the findings highlight the importance of targeted policies and interventions to address TB status concealment within specific demographic and socio-economic groups. Tailoring awareness campaigns, involving community leaders, and providing equal access to healthcare services can collectively contribute to reducing TB status concealment and improving TB control in the population.

### Limitations of the study

The present study offers valuable insights into the factors associated with TB status concealment intentions among individuals aged 10–49 years in seven SSA countries. However, it is essential to acknowledge certain limitations that may impact the interpretation and generalisability of the findings. One primary limitation of this study is its cross-sectional design, which precludes us from establishing causal relationships between the identified factors and TB status concealment intentions. To better understand the temporal relationships and determine the direction of associations, longitudinal studies would be more suitable. Additionally, the use of self-reported data on TB status concealment intentions may introduce social desirability bias. Respondents might be inclined to provide socially acceptable responses, leading to potential underestimation or overestimation of the practice. Future research could benefit from incorporating objective measures or alternative data sources to validate self-reported information. Furthermore, the DHS data utilised in this study were limited to individuals aged 10–49 years, which might not fully represent TB status concealment intentions among younger or older age groups. Including a broader age range in future studies would enhance the generalisability of the results. Another notable limitation is the absence of specific cultural variables in the analysis, which might be strongly associated with TB concealment intentions. Cultural beliefs, norms, and practices related to TB could significantly impact disclosure behaviours. Another significant limitation of this study pertains to the use of datasets characterised by substantial temporal gaps between their collection dates and the current timeframe. The disparity in the dataset timelines may substantially affect the validity and relevance of the conclusions drawn from this study, as well as the proposed recommendations. This discrepancy may raise concerns about the applicability of findings to the present context, given the potential shifts in social, economic, and health-related dynamics over time, which might not be adequately captured by the data from dissimilar timeframes.

Future research should consider incorporating cultural factors to provide a more comprehensive understanding of TB status concealment. Moreover, despite adjusting for several potential confounding variables, other unmeasured factors may influence TB status concealment. Unaccounted variables might lead to residual confounding and affect the observed associations. Further studies employing a more extensive set of covariates could mitigate this limitation. Additionally, the data utilised in this study were obtained from specific regions or countries, limiting the generalisability of the findings to other populations or settings. Researchers should exercise caution when applying these results to different contexts and consider conducting similar studies in diverse populations.

Despite these limitations, the current study contributes valuable insights to the understanding of TB status concealment intentions. By acknowledging these challenges, we hope to encourage future research that addresses these limitations and further advances our understanding of the complex factors influencing TB status concealment intentions. Overall, these findings can serve as a foundation for targeted interventions and policy development to promote open communication and enhance TB control strategies.

## Conclusion

In this study, we found that over a quarter of the sample (28%) had intentions to conceal the TB status of their family members. Through multivariate model analysis, we identified several associations that contribute to our understanding of intentions to conceal the TB status of family members. Age emerged as a critical factor, with individuals aged 20–29, 30–39, and 40–49 being less likely to conceal the TB status of their family members compared to those aged 10–19. This suggests that as individuals grow older, they may become more aware of the importance of timely diagnosis and treatment for TB, leading to greater openness in disclosing the TB status of family members. Notably, the sex of participants did not significantly influence TB status concealment intentions, indicating that this practice is not particularly gender-dependent within the family unit. Regarding marital status, individuals living together were less likely to conceal the TB status of their family members compared to those who were never married, while widowed individuals showed a higher likelihood of concealment. These findings suggest that the type of marital arrangement may influence the disclosure intentions of TB status, potentially due to differences in family dynamics and support systems. The place of residence also played a role, with individuals in rural areas having a lower likelihood of TB status concealment compared to urban areas. This may be attributed to the closer-knit social networks and a stronger sense of community in rural settings, which fosters open communication about health conditions, including TB. Educational level was strongly associated with TB status disclosure, with higher educational attainment being linked to a greater likelihood of openly sharing the TB status of family members. This can be attributed to better health literacy and understanding of the importance of early detection and treatment among individuals with higher education levels. Religion also showed significant associations, with individuals following the Islamic faith being more likely to conceal the TB status of family members compared to Christians, while individuals of other religions were less likely to conceal. This highlights the need to address specific religious beliefs related to TB in targeted awareness campaigns and educational programs. Furthermore, individuals who read newspapers almost every day were more likely to conceal the TB status of their family members, which may indicate the presence of unique communication dynamics within this group that require further investigation. Regarding wealth status, individuals with greater financial resources had a lower likelihood of concealing the TB status of their family members compared to those with lower wealth. This suggests that improved access to healthcare facilities and resources, as well as better health awareness and education, may encourage individuals with higher financial resources to be more open about the TB status of their family members. Finally, the country of residence demonstrated significant associations with TB status concealment intentions, indicating that cultural and contextual factors play a role. For instance, individuals in Ghana, Lesotho, Malawi, Namibia, and Sao-Tome and Principe had higher odds of concealing the TB status of family members compared to Eswatini.

In conclusion, our study sheds light on the diverse factors influencing TB status concealment within families. These findings underscore the importance of targeted public health policies and interventions that consider demographic, socio-economic, and cultural factors to promote open communication about TB and enhance TB control strategies. Policymakers should focus on increasing awareness and understanding of TB, particularly among specific age groups, education levels, and religious affiliations. Addressing socio-cultural contexts, promoting health literacy, and engaging community leaders are essential steps in fostering a supportive environment for TB disclosure and management.

## Data Availability

All data generated or analysed during this study are included in this published article.

## Abbreviations

aOR: Adjusted Odds Ratio
CI: Confidence Interval
DHS: Demographic and Health Surveys
EA: Enumeration Areas
OR: Odds Ratio
SSA: Sub-Saharan Africa
TB: Tuberculosis
WHO: World Health Organisation
